# Systemic inflammation in chronic obstructive pulmonary disease: a population-based study

**DOI:** 10.1186/1465-9921-11-63

**Published:** 2010-05-25

**Authors:** Francisco Garcia-Rio, Marc Miravitlles, Joan B Soriano, Luis Muñoz, Enric Duran-Tauleria, Guadalupe Sánchez, Víctor Sobradillo, Julio Ancochea, EPI-SCAN Steering Committee

**Affiliations:** 1 Pneumology Service, Hospital Universitario La Paz, IdiPAZ, Madrid, Spain; 2 Fundació Clinic, Institut d'Investigacions Biomèdiques August Pi I Sunyer (IDIBAPS), Hospital Clínic, Barcelona, Spain; 3 Fundación Caubet-CIMERA Illes Balears, Bunyola, Illes Balears, and CIBER de Enfermedades Respiratorias, Spain; 4 Pneumology Department, Hospital Reina Sofía, Córdoba, Spain; 5 IMIM/CREAL Barcelona, Spain; 6 Medical Department, GlaxoSmithkline S.A., Madrid, Spain; 7 Pneumology Department, Hospital de Cruces, Bilbao, Spain; 8 Pneumology Department, Hospital La Princesa, Madrid, Spain

## Abstract

**Background:**

Elevated circulating levels of several inflammatory biomarkers have been described in selected patient populations with COPD, although less is known about their population-based distribution. The aims of this study were to compare the levels of several systemic biomarkers between stable COPD patients and healthy subjects from a population-based sample, and to assess their distribution according to clinical variables.

**Methods:**

This is a cross-sectional study design of participants in the EPI-SCAN study (40-80 years of age). Subjects with any other condition associated with an inflammatory process were excluded. COPD was defined as a post-bronchodilator FEV_1_/FVC < 0.70. The reference group was made of non-COPD subjects without respiratory symptoms, associated diseases or prescription of medication. Subjects were evaluated with quality-of-life questionnaires, spirometry and 6-minute walk tests. Serum C-reactive protein (CRP), tumor necrosis factor (TNF)-α, interleukins (IL-6 and IL-8), alpha1-antitrypsin, fibrinogen, albumin and nitrites/nitrates (NOx) were measured.

**Results:**

We compared 324 COPD patients and 110 reference subjects. After adjusting for gender, age, BMI and tobacco consumption, COPD patients showed higher levels of CRP (0.477 ± 0.023 vs. 0.376 ± 0.041 log mg/L, p = 0.049), TNF-α (13.12 ± 0.59 vs. 10.47 ± 1.06 pg/mL, p = 0.033), IL-8 (7.56 ± 0.63 vs. 3.57 ± 1.13 pg/ml; p = 0.033) and NOx (1.42 ± 0.01 vs. 1.36 ± 0.02 log nmol/l; p = 0.048) than controls. In COPD patients, serum concentrations of some biomarkers were related to severity and their exercise tolerance was related to serum concentrations of CRP, IL-6, IL-8, fibrinogen and albumin.

**Conclusions:**

Our results provide population-based evidence that COPD is independently associated with low-grade systemic inflammation, with a different inflammatory pattern than that observed in healthy subjects.

## Background

Chronic obstructive pulmonary disease (COPD) is associated with important extrapulmonary manifestations, including weight loss, skeletal muscle dysfunction, cardiovascular disease, depression, osteoporosis, reduced exercise tolerance, and poor health status [1,2]. Although the pathobiologyof COPD has not been fully determined, systemic inflammation has been implicated in the pathogenesis of the majority of these systemic effects [3], to the point that some authors have suggested that COPD is a part of a chronic systemic inflammatory syndrome [4].

The association between systemic inflammation and COPD has mostly been evaluated in highly selected patient samples, which have shown activation of circulating inflammatory cells and increased levels of proinflammatory cytokines and acute-phase reactants as well as increased oxidative stress [5-7]. The limitations derived from the small size and partial scope of most of these studies led to the completion of a meta-analysis, which compiled the main current evidence supporting the presence of systemic inflammation in stable COPD patients [8]. Nevertheless, there were remarkable differences in the selection of subjects and the definitions of COPD employees were neither homogeneous nor adapted to current guidelines [9]. In the population-based studies included in this analysis, COPD diagnosis was assumed in participants in the lowest quartile of predicted FEV_1_, and those subjects in the highest quartile of predicted FEV_1 _were taken as controls. The controversy has been reinforced by another recent meta-analysis that did not find statistically significant differences in either serum C-reactive protein (CRP) or tumour necrosis factor (TNF)-α concentrations between healthy subject groups and any of the COPD stages [10].

In contrast, an inverse association between higher levels of circulating inflammation-sensitive proteins, including CRP, interleukin (IL)-6 and alpha-1 antitrypsin (A1AT), and lower spirometric values has been described in several samples of middle-aged to older general population [11-13]. Moreover, it has recently been reported that increased serum levels of CRP are associated with an increase risk of developing COPD in a population-based sample of smokers [14].

In the population-based Epidemiologic Study of COPD in Spain (EPI-SCAN) we have compared serum levels of several biomarkers between stable COPD patients and healthy subjects trying to analyse the contribution of possible confounding factors to the development of systemic inflammation. We selected the following biomarkers: CRP, TNF-α, IL-6, IL-8, alpha-1 antitrypsin (A1AT), fibrinogen, albumin and nitrites/nitrates (NOx), because they have been more widely studied in COPD and they have shown some relationship with either its prognosis and/or the development of cardiovascular complications. We have also evaluated the relation between systemic biomarkers and pulmonary function, exercise tolerance and health-related quality of life in COPD patients derived from the general population.

## Methods

### Study design and participants

The present study is part of the EPI-SCAN study, a multicentre, cross-sectional, population-based, observational study conducted at 11 sites throughout Spain [15,16]. The final population recruited was formed by 4,274 non-institutionalized participants from 40-80 years old. The study was approved by the corresponding ethics committees and all participants gave written informed consent.

In accordance with current GOLD guidelines, COPD was defined by a postbronchodilator FEV_1_/FVC ratio < 0.70 [9]. COPD severity was determined by the GOLD criteria and the BODE index [9,17]. Subjects with a postbronchodilator FEV_1_/FVC ratio ≥ 0.70 were considered not to have COPD.

All participants classified as COPD were selected for the systemic biomarker analysis. To avoid excessive testing of the non-COPD study population, an equal number of non-COPD subjects were consecutively selected in each centre. Exclusion criteria for this analysis included a previous diagnosis of acute myocardial infarction, angina, congestive heart failure, cancer, hepatic cirrhosis, chronic renal failure, rheumatoid arthritis or any other systemic inflammatory disease. In addition, specific exclusion criteria from the non-COPD cohort were any respiratory symptoms as per the European Coal and Steel Community (ECSC) questionnaire, any associated concomitant disease, and regularly prescribed medications. The reference group obtained after applying these selection criteria was considered to be of healthy subjects.

### Procedures

Fieldwork and all methods have been described previously [15,16]. Self-reported exposure was identified initially through a questiondeveloped for the European Community Respiratory Health Survey: "Have you ever worked in a job which exposed you to vapors, gas, dust, or fumes?" The question was followedby a list of 23 individual exposures considered a priori risk factors for COPD, subsequently grouped into three categories: biological dusts, mineral dusts and gases or fumes. Baseline dyspnea was assessed by the Modified Medical Research Council (MMRC) scale, and subjects completed the ECSC questionnaire of respiratory symptoms, the London Chest Activity of Daily Living (LCADL) scale, the EQ-5D questionnaire and the St. George's Respiratory Questionnaire.

Blood samples were collected using standardized procedures and stored at -80°C. Samples were shipped to a single laboratory (Hospital Clinic, Barcelona) for centralized analysis approximately every 2 months. TNF-α, IL-6 and IL-8 were determined in duplicate with a high sensitivity enzyme-linked immunosorbent assay (Biosource, Nivelles, Belgium) with lower detection limits of 3 pg/ml for total TNF-α, 2 pg/ml for IL-6 and 0.7 pg/ml for IL-8. The intra-assay coefficients of variation were 3.7% for TNF-α, 2.2% for IL-6 and 2.3% for IL-8. C-reactive protein (CRP) was assessed by latex-enhanced immunonephelometry (Siemens, Dublin, Ireland) with a lower detection limit of 0.4 mg/l and an intra-assay coefficient of variation of 1.2%. Alpha-1 antitrypsin (A1AT) was measured by a particle-enhanced immunonephelometry (Siemens, Malburg, Germany), with detection limits ranged from 0.0095 to 0.3040 g/l and an intra- and interassay variability or 3.9% and 2.0%, respectively.

Albumin levels were estimated by the bromocresol green method (Siemens, Dublin, Ireland), with a detection limits from 10 to 60 g/l and an intra-assay coefficient of variation of 1.5%. Fibrinogen was assessed using a coagulation analyzer (Roche, Mannheim, Germany) according to the Clauss method and calculated from ethylenediamine tetra-acetic acid to citrate plasma values. The detection range was 0.5 to 12.0 g/L and the intra-assay variability 2.8%. Nitrites and nitrates (NOx) were determined by a chemiluminescence detector in an NO analyser (Sievers Instruments, Inc., Boulder, CO, USA). The lower detection limit was 1 pmol and the intra-assay coefficient of variation was 10%.

Baseline and post-bronchodilator spirometries were performed at each site using the same equipment according to current recommendations [18]. The predicted values used were those of the Spanish reference population [19]. A 6-min walk test was performed twice, with an interval between testing of 30 minutes, according to the ATS guidelines [20].

### Analysis

Variables are presented as a percentage, mean ± SD or median (interquartile range) as required depending on their distribution. Statistical analysis was performed with SPSS 14.0 for Windows (SPSS, Inc., Chicago, IL) and with SAS statistical package (version 9.1, Cary, NC). A two-sided p value < 0.05 was considered statistically significant.

Pearson's chi-square test, Mann-Whitney U test or Student's t test were used for two-group comparisons, depending on data distribution. The effect of the possible confounding factors was assessed using generalised linear model analysis [21]. In this analysis, a logarithmic transformation was used in those variables to reduce their skewness. We constructed a multivariate model, including group and gender as fixed factors and age, BMI and smoking history as a dichotomous variable (≥ 10 pack-years, yes/no) as covariates. The link function used was the identity. For each systemic biomarker, we chose the normal distribution because it was more fitting than inverse Gaussian or gamma distribution, according to the plausibility criteria, Pearson's chi-square and analysis of deviance. Comparisons by differing severity within the COPD group were performed using ANOVA analysis, with post-hoc analysis by the Bonferroni test. In the COPD group, the correlations between the serum levels of systemic biomarkers and the clinical and functional parameters were estimated using Pearson's linear bivariate correlation coefficient.

Data are presented according to current recommendations for observational studies in epidemiology (STROBE).

## Results

**Figure 1:**
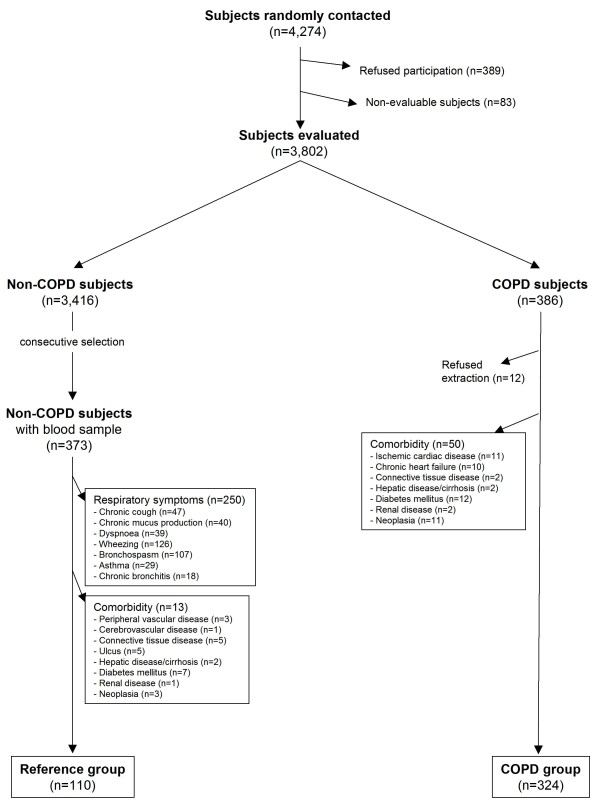
**Flow-chart for the constitution of study groups**.

A total of 3,802 subjects were evaluated. From 386 subjects identified with COPD according to GOLD, 12 refused blood extraction and 50 were excluded due to evidence of comorbidity, leaving 324 subjects in the COPD group for analysis. Of 373 consecutively-selected subjects without COPD, 250 were excluded due to respiratory symptoms and 13 for evidence of comorbidity, rendering 110 subjects in the control group (Figure 1).

Participant characteristics are described in Table 1. In comparison with the reference group, there were more men and smokers, of greater smoking intensity, who were older, with higher body mass index in the COPD group. There was a wide range of COPD severity in our cohort, although only 23% of these patients were taking inhaled corticosteroids. Table 2 shows the occupational exposure characteristics of the patients included in the COPD group.

**Table 1:** General characteristics of the study groups.

	*COPD group* *(n = 324)*	***Reference group*** *(n = 110)*	***p***
Male gender	241 (74%)	51 (46%)	< 0.0001

Age (years)	64 (10)	55 (10)	0.0001

Smoking status			< 0.0001
Never smoker	67 (21%)	66 (60%)	
Former smoker	138 (43%)	30 (27%)	
Current smoker	119 (37%)	14 (13%)	

Smoking exposure (pack-years)	40 (25-55)	10 (5-30)	< 0.0001

Body mass index (Kg/m^2^)	27.9 (4.8)	26.1 (3.4)	0.001

Education level			0.130
Less than primary school	53 (16%)	9 (8%)	
Primary school	120 (37%)	41 (37%)	
Secondary school	84 (26%)	40 (36%)	
University degree	62 (19%)	20 (18%)	

Current treatment			
Short-acting beta-agonist	57 (18%)	0	0.0001
Long-acting beta-agonist	68 (21%)	0	0.0001
Anticholinergic	52 (16%)	0	0.0001
Methylxantines	7 (2%)	0	0.127
Inhaled corticosteroids	75 (23%)	0	0.0001

Pulmonary function			
FVC (L)	3.34 (1.00)	3.96 (1.12)	< 0.0001
FVC (% of predicted)	99 (22)	119 (16)	< 0.0001
FEV_1 _(L)	2.03 (0.67)	3.13 (0.88)	< 0.0001
FEV_1 _(% of predicted)	77 (19)	115 (15)	< 0.0001
FEV_1_/FVC	0.61 (0.08)	0.79 (0.05)	< 0.0001
Postbronchodilator FVC (L)	3.53 (1.01)	3.95 (1.10)	< 0.0001
Postbronchodilator FVC (% of predicted)	105 (21)	119 (14)	< 0.0001
Postbronchodilator FEV_1 _(L)	2.18 (0.69)	3.19 (0.88)	< 0.0001
Postbronchodilator FEV_1 _(% of predicted)	82 (20)	117 (14)	< 0.0001
Postbronchodilator FEV_1_/FVC	0.62 (0.08)	0.81 (0.05)	< 0.0001
Distance walked in 6 minutes (m)	450 (122)	514 (108)	< 0.0001

BODE index score			< 0.0001
Quartile 1 (0-2)	282 (90%)	110 (100%)	
Quartile 2 (3-4)	19 (6%)	0	
Quartile 3 (5-6)	10 (3%)	0	
Quartile 4 (7-10)	2 (0.6%)	0	

EQ-5D questionnaire			
VAS score	75 (60-85)	85 (80.0-93.8)	< 0.0001
Utility score	0.91 (0.83-1.0)	1.0 (1.0-1.0)	< 0.0001
SGRQ			
Total	16.7 (6.2-28.6)	1.3 (0.0-3.3)	< 0.0001
Symptoms	19.6 (8.8-41.2)	4.3 (0.0-9.5)	< 0.0001
Activity	23.6 (6.0-47.7)	0.0 (0.0-0.0)	< 0.0001
Impact	7.6 (1.6-19.5)	0.0 (0.0-0.0)	< 0.0001
LCADL scale	15 (14-17)	15 (15-15)	0.003

Values are mean (SD) or median (interquartile range) depending on the distribution. Abbreviations: FVC = forced vital capacity; FEV_1_= forced expiratory volume in 1 second; SGRQ = St George Respiratory Questionnaire; LCADL = London Chest Activity of Daily Living. Comparisons between groups by U-Mann-Whitney test or t-Student test depending on the distribution.

**Table 2:** Occupational exposure characteristics of COPD patients by smoking status.

	***Never smoker***	***Former smoker***	***Current smoker***	***p***
Subjects, n	67	138	119	
Self-reported exposure to vapors, gases, dusts or fumes Job exposure	27 (40.3%)	54 (39.1%)	49 (41.2%)	0.945
Biological dusts	15 (22.4%)	56 (40.6%)	50 (42.0%)	0.017
Mineral dusts	24 (35.8%)	48 (34.8%)	37 (31.1%)	0.752
Gases or fumes	33 (49.3%)	55 (39.9%)	48 (40.3%)	0.398

Comparisons between groups by chi-square test

**Figure 2:**
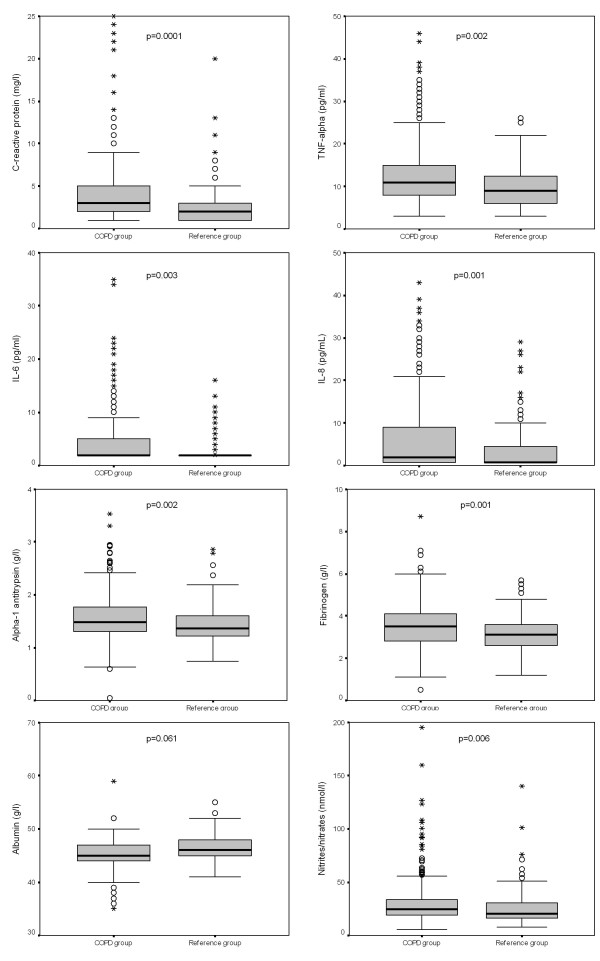
**Box-and-whisker plots of the systemic biomarker crude distribution in COPD and reference groups**. The top of the box represents the 75 th percentile, the bottom of the box represents the 25 th percentile, and the line in the middle represents the 50 th percentile. The whiskers represent the highest and lowest values that are not outliers or extreme values. Outliers (values that are between 1.5 and 3 times the interquartile range) and extreme values (values that are more than 3 times the interquartile range) are represented by circles and asterisks beyond the whiskers. Abbreviations: TNF = tumor necrosis factor; IL = interleukin. Comparisons between groups by U-Mann-Whitney test or t-Student test depending on the distribution.

The crude comparison of serum level biomarkers showed that COPD participants had higher concentrations of CRP, TNF-α, IL-6, IL-8, alpha-1 antitrypsin, fibrinogen and nitrites/nitrates than control subjects (Figure 2). On the contrary, albumin concentration was non-significantly decreased (p = 0.061).

**Figure 3:**
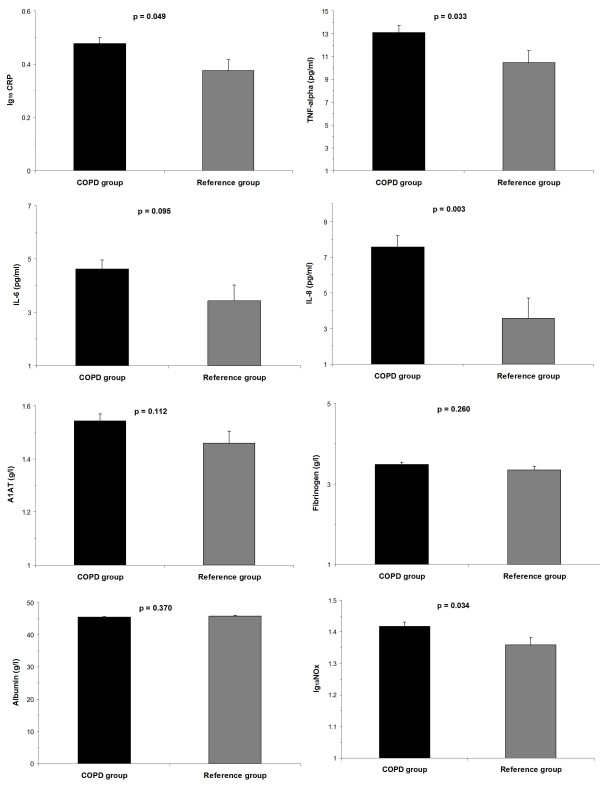
**Serum concentrations of systemic biomarkers in COPD patients and control subjects**. Data are presented as mean adjusted for age, sex, pack-years of smoking and body-mass index (standard error of the mean). A logarithmic transformation was used for CRP and NO_x_. Abbreviations: CRP = C-reactive protein; TNF = tumor necrosis factor; IL = interleukin; A1AT = alpha-1 antitrypsin; NO_x _= nitrites/nitrates.

Table 3 shows the estimates obtained from generalized linear models with gender, age, BMI, pack-years and group as dependent variables. After adjusting for these covariates, group dependence was retained for CRP, TNF-α, IL-8 and nitrites/nitrates, with a positive effect on their serum concentrations. After adjustment for gender, age, BMI and pack-years, COPD participants presented higher levels of log CRP (mean ± mean standard error) (0.477 ± 0.023 vs. 0.376 ± 0.041 log mg/L, p = 0.049), TNF-α(13.12 ± 0.59 vs. 10.47 ± 1.06 pg/mL, p = 0.033), IL-8 (7.56 ± 0.63 vs. 3.57 ± 1.13 pg/ml; p = 0.033) and nitrites/nitrates (1.42 ± 0.01 vs. 1.36 ± 0.02 log nmol/l; p = 0.048). No differences for adjusted levels of alpha-1 antitrypsin, IL-6, fibrinogen or albumin were found between COPD and reference subjects (Figure 3).

**Table 3:** Significance of each multivariate model to estimate systemic biomarkers*.

***Biomarker***	***Parameter***	***Coefficient (SE)***	***Wald 95% CI***	*p-value*
C-reactive protein†	Intercept	-0.44 (0.173)	-0.785- -0.103	0.011
	Age	0.005 (0.002)	0.001-0.009	0.012
	BMI	0.019 (0.004)	0.011-0.028	0.001
	Smoker	0.036 (0.155)	-0.268-0.340	0.816
	Gender	-0.004 (0.044)	-0.089-0.083	0.936
	COPD group	0.101 (0.049)	0.004-0.198	0.041
				
TNF-alpha	Intercept	6.306 (4.301)	-2.151-14.763	0.143
	Age	0.062 (0.049)	-0.034-0.158	0.207
	BMI	0.059 (0.110)	-0.157-0.276	0.590
	Smoker	0.810 (3.434)	-5.942-7.562	0.814
	Gender	-0.989 (1.088)	-3.128-1.150	0.364
	COPD group	2.668 (1.250)	0.211-5.125	0.033
				
IL-6	Intercept	3.420 (2.477)	-1.450-8.290	0.168
	Age	0.025 (0.028)	-0.030-0.081	0.367
	BMI	0.017 (0.063)	-0.108-0.142	0.787
	Smoker	-0.597 (1.934)	-4.399-3.204	0.758
	Gender	-1.525 (0.619)	-2.741- -0.309	0.014
	COPD group	1.194 (0.713)	-0.207-2.595	0.095
				
IL-8	Intercept	9.436 (4.712)	0.173-18.700	0.046
	Age	-0.007 (0.053)	-0.112-0.098	0.892
	BMI	-0.214 (0.121)	-0.451-0.024	0.078
	Smoker	2.951 (3.672)	-4.267-10.169	0.422
	Gender	0.277 (1.175)	-2.034-2.587	0.814
	COPD group	3.995 (1.350)	1.342-6.648	0.003
				
Alpha-1 antitrypsin	Intercept	1.307 (0.184)	0.947-1.668	< 0.001
	Age	0.003 (0.002)	-0.001-0.007	0.153
	BMI	0.001 (0.005)	-0.011-0.008	0.785
	Smoker	0.069 (0.143)	-0.351-0.213	0.630
	Gender	0.003 (0.046)	-0.087-0.093	0.945
	COPD group	0.084 (0.053)	-0.019-0.187	0.112
				
Fibrinogen	Intercept	0.484 (0.416)	-0.335-1.302	0.246
	Age	0.030 (0.005)	0.020-0.039	0.001
	BMI	0.021 (0.011)	0.000-0.042	0.050
	Smoker	-0.087 (0.325)	-0.726-0.551	0.788
	Gender	0.344 (0.104)	0.139-0.549	0.001
	COPD group	0.134 (0.119)	-0.100-0.369	0.260
				
Albumin	Intercept	50.071 (1.165)	47.781-52.361	0.001
	Age	-0.066 (0.013)	-0.092- -0.040	0.001
	BMI	0.028 (0.030)	-0.031-0.087	0.350
	Smoker	-0.083 (0.911)	-1.873-1.707	0.928
	Gender	-0.759 (0.290)	-1.329- -0.188	0.009
	COPD group	-0.299 (0.333)	-0.955-0.356	0.370
				
Nitrites/nitrates†	Intercept	1.636 (0.097)	1.445-1.828	0.001
	Age	-0.001 (0.001)	-0.003-0.001	0.35
	BMI	-0.004 (0.002)	-0.009-0.000	0.116
	Smoker	-0.007 (0.076)	-0.157-0.143	0.926
	Gender	-0.079 (0.024)	-0.127- -0.031	0.001
	COPD group	0.059 (0.028)	0.004-0.114	0.034

* Main effects of factors and covariates included in the generalized linear model analysis. Smoker was defined as current or former smoker of > 10 packs-year (yes/no). COPD group effect was estimated versus reference group. † Parameter with logarithmic transformation.

Serum concentrations of several systemic biomarkers were mostly higher in severe COPD than in moderate or mild COPD. Of interest, these differences with biomarker concentrations were not concordant with severity assessed by GOLD and the BODE index (Tables 4 and 5), and the biomarkers most consistent for the severity discrimination were CRP, IL-6 and nitrites/nitrates.

**Table 4:** Distribution of systemic biomarkers by severity of COPD according to GOLD criteria *.

	***Mild COPD*** ***(n = 177)***	***Moderate COPD*** *(n = 128)*	***Severe COPD*** *(n = 19)*	*p*
Male (%)	67.8	82.0	84.2	0.012
Age (yr)	62 (54-70)	67 (58-73) †	70 (66-74) †	0.003
BMI (Kg/m^2^)	27.4 (4.7)	28.5 (4.7)	28.7 (6.4)	0.107
Smoking status				0.135
Never smoker	37.6	37.5	21.1	
Former smoker	37.9	46.1	63.2	
Current smoker	24.3	16.4	15.8	
Smoking exposure (packs-year)	30 (20-47)	45 (30-60) ‡	40 (22-54)	0.001
C-reactive protein (mg/l)	3.0 (2.0-5.0)	3.0 (2.0-6.0)	2.0 (2.0-12.0) ‡	0.007
TNF-alpha (pg/ml)	10.0 (7.0-14.0)	11.0 (8.0-18.0) †	11.0 (5.0-14.0)	0.017
IL-6 (pg/ml)	1.9 (1.9-3.0)	1.9 (1.9-7.0)	3.0 (1.9-16.0) †	0.008
IL-8 (pg/ml)	2.0 (0.7-7.8)	2.0 (0.7-10.0)	7.0 (0.9-11.0)	0.323
Alpha-1 antitrypsin (g/l)	1.47 (1.27-1.72)	1.47 (1.32-1.73)	1.59 (1.38-1.83)	0.706
Fibrinogen (g/l)	3.46 (1.04)	3.63 (1.11)	3.73 (1.17)	0.305
Albumin (g/l)	45.36 (2.54)	45.41 (3.03)	44.67 (3.51)	0.571
Nitrites/nitrates (nmol/l)	26.3 (20.8-38.1)	23.3 (19.1-31.2)	27.1 (17.7-61.8)	0.048

* Values are mean (SD) or median (interquartile range) depending on the distribution. Abbreviations: TNF = tumour necrosis factor; IL = interleukin. Comparisons between groups by ANOVA with Bonferroni test: † p < 0.05 vs. mild COPD; ‡ p < 0.01 vs. mild COPD; p < 0.05 vs. moderate COPD; § p < 0.01 vs. moderate COPD.

**Table 5:** Comparison of systemic biomarkers by severity of COPD according to quartiles of BODE index*.

	***Quartile 1*** *(n = 282)*	***Quartile 2*** *(n = 19)*	***Quartiles 3-4*** *(n = 12)*	***p***
Male (%)	73.8	84.2	58.3	0.279
Age (yr)	60 (55-71)	72 (69-75) †	66 (61-72)	0.007
BMI (Kg/m^2^)	27.7 (4.3)	29.9 (8.0)	31.1 (8.2) †	0.011
Smoking status				0.303
Never smoker	38.3	31.6	16.7	
Former smoker	41.1	57.9	50.0	
Current smoker	20.6	10.5	33.3	
Smoking exposure (packs-year)	40 (25-50)	50 (23-85)	50 (32-59)	0.050
C-reactive protein (mg/dl)	3.0 (2.0-5.0)	3.0 (2.0-6.0)	4.5 (2.0-24.7) ‡§	0.0001
TNF-alpha (pg/ml)	10.0 (7.0-14.5)	11.0 (8.5-16.0)	12.0 (7.7-15.0)	0.992
IL-6 (pg/ml)	1.9 (1.9-4.0)	3.0 (1.9-12.0)	2.5 (1.9-12.0)	0.032
IL-8 (pg/ml)	2.0 (0.7-8.0)	7.0 (4.0-13.0)	10.5 (0.7-25.2) †	0.004
Alpha-1 antitrypsin (g/l)	1.46 (1.29-1.69)	1.56 (1.24-1.72)	1.69 (1.56-1.84)	0.444
Fibrinogen (g/l)	3.54 (1.06)	3.66 (1.29)	3.93 (1.14)	0.446
Albumin (g/l)	45.39 (2.73)	44.06 (3.49)	45.75 (2.34)	0.122
Nitrites/nitrates (nmol/l)	24.9 (19.7-34.2)	29.5 (20.1-35.4)	22.4 (16.4-83.2) †	0.030

* Values are mean (SD) or median (interquartile range) depending on the distribution. Abbreviations: TNF = tumour necrosis factor; IL = interleukin. Comparisons between groups by ANOVA with Bonferroni test: † p < 0.05 vs. quartile 1, ‡ p < 0.01 vs. quartile 1; § p < 0.01 vs. quartile 2.

**Figure 4:**
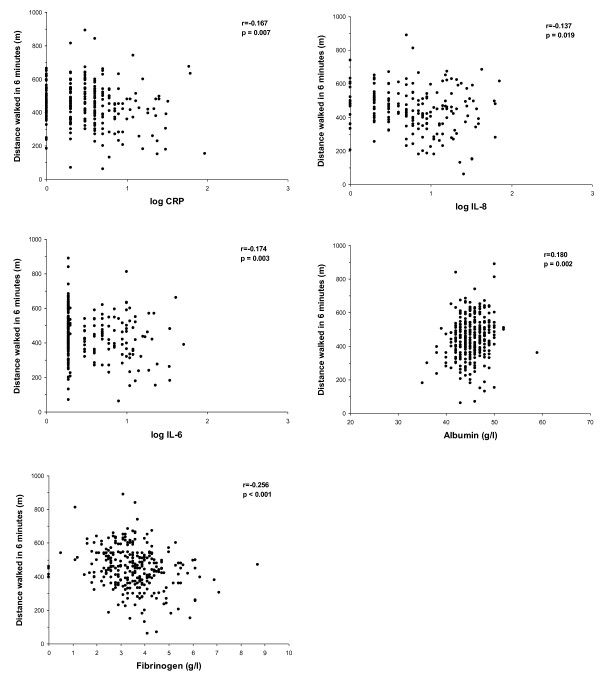
**Relationship between serum concentrations of biomarkers and distance walked in 6-minutes in COPD patients**. Abbreviations: CRP = C-reactive protein; IL = interleukin; r = Pearson's linear bivariate correlation coefficient.

In COPD participants, a relationship between systemic biomarker concentrations and health status scores was found. Dyspnea intensity, assessed by the MMRC, was weakly related to CRP (r = 0.133, p = 0.027) and to fibrinogen concentrations (r = 0.131, p = 0.021). A weak relationship between the symptoms domain of the SGRQ and the IL-8 serum concentration was noted (r = 0.112, p = 0.049), while the activity domain was related with CRP (r = 0.164, p = 0.006), IL-6 (r = 0.117, p = 0.039), fibrinogen (r = 0.158, p = 0.006) and albumin (r = -0.140, p = 0.014). Indeed, CRP level was also weakly related to the visual analogue scale score (r = -0.146, p = 0.015) and utility score in the EQ-5D (r = -0.121, p = 0.045). Biomarker serum concentrations also showed a weak relationship with the functional characteristics of COPD patients. Post-bronchodilator FEV_1 _was inversely related to CRP (r = -0.142, p = 0.018) and to IL-6 (r = -0.190, p = 0.023). In the same way, we found a weak relationship between exercise tolerance and serum concentrations of CRP (r = -0.167, p = 0.007), IL-6 (r = -0.174, p = 0.003), IL-8 (r = -0.137, p = 0.019), fibrinogen (r = -0.256, p < 0.001) and albumin (r = 0.180, p = 0.002) (Figure 4).

## Discussion

This study provides population-based evidence that stable COPD patients have a pro-inflammatory state, with increased circulating levels of many inflammatory cytokines and acute-phase reactants. In addition to the contribution of previously-recognized factors such as age, gender, BMI or smoking, COPD constitutes an independent factor for the elevation of many of the analyzed systemic biomarkers, which in the case of CRP, TNF-alpha, IL-6 and NOx is also dependent on severity. Finally, baseline inflammatory markers show a relation with some domains of health-related quality of life, airflow limitation and exercise tolerance.

### Confounding factors

To adequately evaluate the effect of COPD on systemic biomarkers, several risk factors associated with COPD should be considered. COPD is an age-related disorder and the normal process of aging appears to be associated with a similar low-grade systemic inflammatory process [16,22]. The importance of gender is given by the fact that females have a more vigorous inflammatory reaction and generate more oxidative stress in the airways than males [23]. Although an abnormal systemic inflammatory reaction is detected in most smokers, it has been demonstrated that some systemic biomarkers remain persistently high after smoking cessation [24], suggesting the contribution of other factors. For this reason, some authors propose to evaluate the impact of tobacco on systemic biomarkers depending on whether a dose threshold (10 pack-years) has been reached [25]. Obesity is associated with low-grade systemic inflammation and it has been suggested that the distribution of body compartments might originate a different behaviour of some inflammatory markers [26,27]. In concordance with previous reports [28], a direct correlation was found between BMI and CRP (r = 0.242, p = 0.0001) in the COPD participants of our study.

### Systemic biomarkers in COPD

After adjusting for possible confounding factors, we report that COPD patients showed higher levels of TNF-α, IL-6, IL-8, CRP and nitrites/nitrates than control subjects. The origin of systemic inflammation in COPD is not completely clear. The hypothesis that systemic inflammation is originated by spill over from the pulmonary compartment has not yet been proven [3]. It has been suggested that some common genetic or constitutional factors may predispose individuals with COPD towards both systemic and pulmonary inflammation [29]. Lung hyperinflation, tissue hypoxia and skeletal muscle and bone marrow alterations have also been implicated in the induction of systemic inflammation [3].

Although an increased production of NO in COPD patients could constitute a host defense mechanism, a high level of NO can also cause injury and thus contribute to the respiratory and systemic features of the disease. In an inflammatory environment, exaggerated production of NO in the presence of oxidative stress may produce the formation of strong oxidizing reactive nitrogen species, such as peroxynitrite, leading to nitration, which provokes inhibition of mitochondrial respiration, protein dysfunction and cell damage [30]. The activation of various heme peroxidases by hydrogen peroxide can promote oxidation of nitrites to intermediates that are capable of nitrating aromatic substratesand proteins [30].

Although the COPD severity classification according to the BODE index shows a great capacity for discriminating among the systemic biomarker levels, as expected from its multicomponent character, the GOLD classification also shows differences in biomarker levels. However, the selection of a small number of severe patients in our population sample may reduce the strength of a possible association between biomarkers and GOLD stage. In some previous studies, the relation between plasma CRP levels and the severity of the disease has already been suggested [5,31]. De Torres and colleagues reported the usefulness of CRP in predicting clinical and functional outcomes in stable COPD, with similar correlation coefficients to those of our study [27].

Nevertheless, one of the major implications of systemic inflammation in COPD is its contribution to a proatherosclerotic state. The relationship between COPD, systemic inflammation, and cardiovascular diseases may be especially relevant as over half of patients with COPD die from cardiovascular causes [32]. A Copenhagen City Heart Study cohort study showed that the incidence of COPD hospitalization and COPD death was higher in individuals with baseline CRP above 3 mg/L, with an absolute 10-yr risk for death of 57% [33]. In fact, it has been suggested that CRP can be considered as the sentinel biomarker [32,33]. Interesting, in our COPD patients, serum CRP levels were related to concentrations of IL-6 (r = 0.333, p < 0.001), IL-8 (r = 0.125, p = 0.039), fibrinogen (r = 0.356, p < 0.001) and A1AT (r = 0.194, p < 0.001).

In our COPD patients, CRP and IL-6 were inversely related to postbronchodilator FEV_1 _(% predicted). However, the contribution of systemic inflammation to lung function decline is less clear. While crossectional studies show that systemic inflammatory markers are inversely related to lung function [6,13,25], a prospective evaluation of lung function decline in a randomly selected population did not identify this negative effect over a 9-year period [34].

Finally, we found that exercise tolerance, as assessed by the distance walked in the 6-minute test was inversely related to serum CRP, IL-6 and IL-8 levels. IL-6 is produced by contracting muscles and released into the blood, acting as an energy sensor. When contracting muscles are low in glycogen, IL-6 gene transcription is increased and IL-6 is released to increase glucose uptake and induce lipolysis [35]. When muscles are exposed to oxidative stress, both IL-6 mRNA and IL-6 protein expression are enhanced [35]. It is known that COPD patients with high plasma levels of CRP had more impaired energy metabolism, increased disability and more distress due to respiratory symptoms than patients with normal CRP levels [35]. Moreover, the relation between serum CRP levels and exercise tolerance seems to be independent of other factors such as age, sex, and smoking history [36]. Whether skeletal muscle dysfunction is a direct consequence of the systemic effects of the COPD or an independent process that contributes to the systemic inflammatory load of the disease, our results indicate that systemic biomarker levels could indirectly reflect the exercise capacity of these patients.

### Strengths and weakness of the study

The strengths of this study include its population-based design, the use of post-bronchodilator spirometry as diagnostic criteria and the detailed characterization of the participants which allowed us to investigate factors associated with circulating biomarker levels. In addition to the post-bronchodilator spirometric criteria confirming the existence of an irreversible obstruction, in our never-smoker COPD patients there was evidence of the existence of a particular exposure history that could support the diagnosis.

Although it has been reported that the fixed FEV_1_/FVC ratio method results in a greater proportion of COPD diagnoses than other alternative methods, especially in the elderly, it still continues to be the criterion established in the GOLD guidelines and is certainly the most widely used in clinical practice. The current debate about LLN as an alternative to the fixed ratio is based more on the opinions of experts [37,38] than on the existence of clear evidence. In this situation, the information provided by the Cardiovascular Health Study is especially relevant, demonstrating that a cohort of elderly subjects classified as ''normal'' using the LLN but abnormal using the fixed ratio were more likely to die and to have a COPD-related hospitalization during an 11-year follow-up [39]. Thus, a fixed FEV_1_/FVC ratio < 0.70 may identify at-risk patients, even among older adults.

There are several potential limitations of our study worth discussing. Firstly, our COPD patient sample does not turn out to be necessarily representative of the COPD regularly seen in clinical practice. Due to the design of the present study, and to avoid the confusion by comorbidities, patients with several associated illnesses were excluded from the analysis. This strategy probably reduces differences between the two study groups, but it assures a stricter evaluation of these. Secondly, there were significant differences between COPD patients and reference subjects for anthropometric characteristics and smoking status. These are consequences of the population-based extraction of our study subjects and are partly a reflection of the fact that some differences are likely manifestations of the disease. Although the statistical model aims to adjust for these possible confounding factors, the existence of some uncontrolled effects cannot be excluded. And finally, this is a cross-sectional study, and inference of causality is not possible. Our results could also be affected by other drawbacks. Of the COPD patients in our study, 23% were using inhaled corticosteroids. This could contribute to the underestimation of the difference in systemic biomarkers between COPD patients and control subjects. Nevertheless, the effect of inhaled corticosteroids on inflammatory biomarkers is still controversial. In patients with moderate to severe COPD, it has been reported that one month of fluticasone did not reduce serum CRP or IL-6 levels [28]. Other chronic conditions, such as chronic heart failure or diabetes, also appear to be associated with a similar low-grade systemic inflammatory process [22]. Several studies have described that these disorders are more frequent in COPD patients and, therefore, might also contribute to their proinflammatory state. Nevertheless, and in order to analyze only the effect of COPD as an independent factor, we have carefully excluded these conditions from the present study.

## Conclusions

This study provides information about the population-based distribution of some systemic biomarkers according to lung function and BODE index, and reinforces the evidence that COPD is independently associated with low-grade systemic inflammation, with a different inflammatory pattern than the one observed in healthy subjects. In addition to its contribution to the extrapulmonary effects of COPD, the intensity of the systemic inflammation is directly related to the poorer quality of life, airflow limitation and exercise intolerance observed in COPD. These results emphasize the importance of carrying out multidimensional evaluations of COPD patients, of interest to understanding the mechanisms involved in COPD development and progression, as well as for the management of individual patients.

## Competing interests

GS is a full-time employee of GlaxoSmithKline, drug manufacturerand sponsor of the study. However, the subject of the studyis epidemiological with no drugs involved. The rest of authorsdo not have any conflict of interest with relation to the contentsof the manuscript.

## Authors' contributions

FGR served as the primary author, reviewing all data, and wrote the article. MM, JBS, LM, EDT, GS, VS and JA developed the study protocol, interpreted study data, contributed to and reviewed drafts of the manuscript, and approved the final version of the manuscript. All authors read and approved the final manuscript. The academic authors vouch for the veracity and completeness of the data and the data analyses. The study sponsor did not place any restrictions with regard to statements made in the final version of the article.

## EPI-SCAN Team

### Scientific Committee

Julio Ancochea. Hospital La Princesa (Madrid)

Carlos Badiola, GlaxoSmithkline (Madrid)

Enric Duran. Institut Municipal d'Investigació Mèdica (IMIM) (Barcelona)

Francisco García Río. Hospital La Paz (Madrid)

Marc Miravitlles. Hospital Clínic (Barcelona)

Luis Muñoz. Hospital Reina Sofía (Córdoba)

Víctor Sobradillo. Hospital de Cruces (Bilbao)

Joan B Soriano. Fundació Caubet-CIMERA Illes Balears (Bunyola, Illes Balears)

### Participating sites and coordinators

Julio Ancochea. Hospital La Princesa (Madrid)

Luis Borderias. Hospital San Jorge (Huesca)

Francisco García-Río. Hospital La Paz (Madrid)

Jaime Martínez. Hospital Central de Asturias (Oviedo)

Teodoro Montemayor. Hospital Virgen de la Macarena (Sevilla)

Luis Muñoz. Hospital Reina Sofía (Córdoba)

Luis Piñeiro. Hospital Xeral Cies (Vigo)

Joan Serra. Hospital General de Vic (Vic, Barcelona)

Juan José Soler-Cataluña. Hospital General de Requena (Requena, Valencia)

Antoni Torres. Hospital Clínic (Barcelona)

José Luis Viejo. Hospital General Yagüe (Burgos)
